# Automated Differentiation of Atypical Parkinsonian Syndromes Using Brain Iron Patterns in Susceptibility Weighted Imaging

**DOI:** 10.3390/diagnostics12030637

**Published:** 2022-03-05

**Authors:** Yun Soo Kim, Jae-Hyeok Lee, Jin Kyu Gahm

**Affiliations:** 1 Department of Information Convergence Engineering, Pusan National University, Busan 46241, Korea; kim8yunsu16@pusan.ac.kr; 2 Department of Neurology, Pusan National University Yangsan Hospital, Pusan National University School of Medicine, Yangsan 50612, Korea; jhlee.neuro@pusan.ac.kr; 3 School of Computer Science and Engineering, Pusan National University, Busan 46241, Korea

**Keywords:** atypical parkinsonian syndromes, brain iron, SWI, radiomic, machine learning

## Abstract

In recent studies, iron overload has been reported in atypical parkinsonian syndromes. The topographic patterns of iron distribution in deep brain nuclei vary by each subtype of parkinsonian syndrome, which is affected by underlying disease pathologies. In this study, we developed a novel framework that automatically analyzes the disease-specific patterns of iron accumulation using susceptibility weighted imaging (SWI). We constructed various machine learning models that can classify diseases using radiomic features extracted from SWI, representing distinctive iron distribution patterns for each disorder. Since radiomic features are sensitive to the region of interest, we used a combination of T1-weighted MRI and SWI to improve the segmentation of deep brain nuclei. Radiomics was applied to SWI from 34 patients with a parkinsonian variant of multiple system atrophy, 21 patients with cerebellar variant multiple system atrophy, 17 patients with progressive supranuclear palsy, and 56 patients with Parkinson’s disease. The machine learning classifiers that learn the radiomic features extracted from iron-reflected segmentation results produced an average area under receiver operating characteristic curve (AUC) of 0.8607 on the training data and 0.8489 on the testing data, which is superior to the conventional classifier with segmentation using only T1-weighted images. Our radiomic model based on the hybrid images is a promising tool for automatically differentiating atypical parkinsonian syndromes.

## 1. Introduction

In neurodegenerative disease, abnormal neuronal cells die rapidly in parts of the nervous system or the entire brain, resulting in loss of brain function, including cognitive and motor abilities. Parkinson’s disease (PD) is the second most common neurodegenerative disorder after Alzheimer’s and is accompanied by motor symptoms such as bradykinesia, tremor, and gait disturbance, making it difficult to conduct daily activities and many nonmotor symptoms such as cognitive impairment, depression, autonomic dysfunction, and sleep disturbance. Atypical parkinsonian syndromes (APSs), comprising of progressive supranuclear palsy (PSP) and a parkinsonian variant of multiple system atrophy (MSA-P), are degenerative diseases that share similar Parkinsonism symptoms and signs with PD [1] but show additional symptoms and different rates of functional deterioration and prognosis [2]. Therefore, the development of methods for distinguishing between PD and APS has clinical significance.

One of the main pathogenesis of PD is iron accumulation in the substantia nigra area of the brain associated with the degeneration of dopaminergic neurons and accumulation of misfolded proteins [3]. According to recent pathological studies, each parkinsonian syndrome has unique topographic patterns of iron distribution in deep brain nuclei, which are influenced by underlying disease pathologies [4,5].

There have been many studies using advanced magnetic resonance images (MRI) to detect the physiological mechanisms underlying PD and to distinguish APS from PD, such as using resting-state functional MRI (fMRI) [6] or diffusion MRI [7], but these approaches are not easy to apply in general clinical practice because they are time consuming and do not guarantee consistent results [8]. In addition, various studies using other modalities, including PET and SPECT, can achieve significant diagnostic relevance with respect to imaging of PD and APS [9,10]. However, there are also some disadvantages of these modalities, such as radiation exposure of CT [11], and obstacles to the clinical application of PET by limited access and high examination costs [12]. These common advanced neuroimaging techniques are summarized in Table 1.

**Figure 1:**
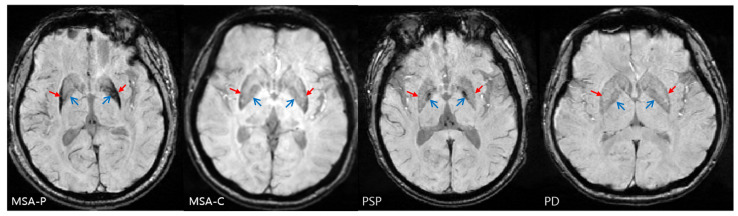
SWI axial view of parkinsonian syndrome patients: parkinsonian variant multiple system atrophy (MSA-P), cerebellar variant multiple system atrophy (MSA-C), progressive supranuclear palsy (PSP), and Parkinson’s disease (PD). Increased iron-related signals in the anterior and medial aspects of the globus pallidus (open arrow) of SWI is a highly specific sign of PSP. For MSA-P, significant accumulation of iron in the lateral aspect of the globus pallidus adjacent to putamen, posterolateral putaminal hypointensity, (closed arrow) and lateral-to-medial gradient appear consistently.

Susceptibility weighted imaging (SWI), a type of iron-sensitive MRI, is frequently used to detect disease-specific patterns of uneven and localized iron concentration in brain regions [13]. Figure 1 shows the sample SWI axial slices of the MSA-P, MSA-C, PSP, and PD. Increases in iron-related signals in the anterior and medial aspects of the globus pallidus of SWI are highly specific markers of PSP. For MSA-P, a significant accumulation of iron is present in the lateral aspect of the globus pallidus adjacent to the putamen. In addition, the posterolateral putaminal hypointensity and lateral-to-medial gradient appear consistently in MSA-P SWI [14]. However, assessing the putaminal hypointensity by focusing only on the signal intensity without accounting the distributional pattern fails to differentiate between MSA-P from PD [15]. A generic and age-related sign of physiological mineralization is slit-like hypointensity along the lateral margin of the putamen or evenly distributed hypointensity throughout the putamen [16]. Therefore, finding a distinctive pattern that distinguishes parkinsonian syndromes besides nonspecific and age-related signs is challenging.

To analyze the regional iron heterogeneity in deep brain nuclei without an expert radiologist, radiomic features provide advanced quantification and classification methodologies based on machine learning algorithms. Radiomics can extract textural features that express the relationship with neighboring voxels, allowing us to analyze the regional iron deposition in the subcortical structures. It is suitable for SWI, where the signal itself cannot be used because the SWI intensities of non-paramagnetic materials, such as white matter (WM) and cerebrospinal fluid (CSF), are modified through the filtered phase mask to emphasize the susceptibility in the image. There is considerable interest in the potential of radiomics for non-invasive biomarkers in different organs and pathologies, including neurodegenerative diseases [17].

Since radiomics is sensitive to changes in image intensities, accurate and robust segmentation of deep gray matter (DGM) nuclei is required. Although manually viewing the image and judging the lesion or progression is highly accurate when performed by an expert radiologist, it has the disadvantages of high time consumption and monetary costs to diagnose large numbers of patients. To overcome these problems, several automated segmentation tools based on T1-weighted (T1w) images have been developed including FreeSurfer [18,19], FMRIB software library (FSL) integrated registration and segmentation tool (FIRST) [20], and others [21]. These techniques have been applied in multiple brain imaging studies for examining volume and shape changes in subcortical brain regions that may be linked to normal aging or neurodegenerative disorders. DGM segmentation is scan–rescan reliable on the same scanning platform and between separate scanning platforms, indicating that these tools may be used in large-scale longitudinal and multisite studies [22,23]. However, if only T1w images are used as atlas-based tools, the segmentation results tend to be inaccurate [24] and do not represent the patient’s hallmarks, because the spatial correspondence of subcortical structures between an abnormal brain and standard atlas is poor and the contrast of DGM in T1w images is insufficient [25]. Therefore, it is necessary to develop a segmentation method that better reflects the distinctive features of each disease using a modality other than T1w.

In this paper, we propose a novel framework that uses the SWI to automatically analyzed the disease-specific patterns of iron accumulation. Our contributions to this study are listed below:

- We proposed a fully automatic framework for the analysis of iron deposition patterns in SWI.
- We developed segmentation that reflects more the contrast of iron accumulation than conventional methods using a hybrid contrast image, which is created by image processing and combining T1w and SWI.
- We designed machine learning classifiers trained using texture-representing features extracted by our segmentation method.
- We demonstrated the improved performance of the machine learning classifier for differentiating APS using our segmentation framework.

The remainder of the paper is organized as follows. In Section 2, we propose an automated framework for SWI segmentation and the radiomic learning model, including hybrid image generation, DGM segmentation, radiomic feature extraction and selection, and machine learning classifier validation. Experimental results are presented in Section 3, wherein the proposed algorithm is validated using the patient datasets. Finally, the main conclusions and discussions are made in Section 4.

## 2. Materials and Methods

**Figure 2:**
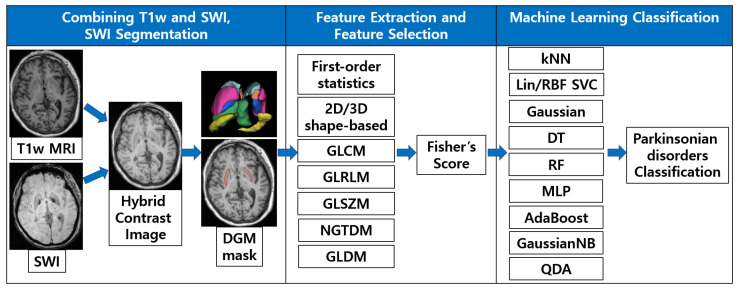
Overall flowchart of combining T1w and SWI, SWI segmentation, feature extraction and selection, and disease classification. We create a hybrid image combining T1w and SWI for iron-reflected DGM segmentation, extract texture representative features, and classify parkinsonian disorders with the significant features selected using various machine learning algorithms.

In this section, we describe the details of our framework that automatically differentiate APS using brain iron patterns in SWI. Figure 2 presents the overall framework of the proposed method. First, a DGM mask using the advantages of both T1w and SWI was obtained by optimally combining preprocessed and registered images. The radiomic features were retrieved from the brain regions of interest (ROI) by adjusting the distance between the neighboring voxels. Thereafter, a machine learning feature selection algorithm was applied to select meaningful features that distinguish the diseases. Finally, various machine learning classifiers were trained and tested using the selected features.

### 2.1. Patients

A total of 34 MSA-P, 21 MSA-C, 17 PSP, and 56 PD patients were enrolled from the Pusan University Yangsan Hospital. The following clinical diagnostic criteria were fulfilled by the patients: PSP diagnosed according to the Litvan criteria [26], MSA according to clinical consensus criteria [27], and PD according to the UK Brain Bank criteria [28]. Movement Disorder Society (MDS) PSP criteria were retrospectively applied to all consecutive patients with PSP. Twelve patients were classified as probable PSP Richardson’s syndrome (PSP-RS) and five were classified as probable PSP with predominant Parkinsonism (PSP-P). Subjects with microvascular lesions discovered from brain MRI were excluded. The Hoehn and Yahr (H&Y) stage and motor examination part of the Unified Parkinson’s Disease Rating Scale (UPDRS III) were used to measure disease severity and motor symptoms. Written and informed consent was obtained from all subjects participating in the study, which was approved by the Pusan National University Institutional Review Board, in accordance with the guidelines of the Helsinki Declaration.

### 2.2. Imaging Acquisition

We obtained the 3D magnetization prepared rapid gradient echo (MPRAGE) axial or sagittal T1w and SWI MRI volumes of 34 MSA-P, 21 cerebellar variants of MSA (MSA-C), 17 PSP, and 56 PD patients from the Pusan National University Yangsan Hospital using protocols approved by the institutional review board. The MRI scans were conducted using a 3.0T MRI scanner (Verio, Siemens, Erlangen, Germany). The T1w data were acquired under the following sequence parameters: echo time (TE) = 2.2 ms, repetition time (TR) = 1900 ms, inversion time (TI) = 900 ms, flip angle (FA) = 9, dimensions = 280 × 320 × 176, and voxel size = 0.75 mm × 0.75 mm × 1 mm. The SWI data were acquired under the following sequence parameters: TE = 20 ms, TR = 28 ms, FA = 15, dimensions = 260 × 320 × 64, and voxel size = 0.6875 mm × 0.6875 mm × 2 mm.

### 2.3. Data Preprocessing and SWI Registration

We performed SWI postprocessing as the first step. Magnitude, high-pass filtered phase images, and the processed SWI data were reconstructed automatically on a workstation (Syngo, Siemens Medical Solution) as a DICOM file format for analysis. Then, we created an initial segmentation mask for T1w to use when creating HC through FreeSurfer reconstruction. Non-parametric non-uniform and intensity normalization (N4ITK) bias-field correction [29] and intensity normalization were applied. We applied intensity normalization to scale the T1w signal intensity to a predefined mean value of 110 in the white matter (WM).

**Figure 3:**
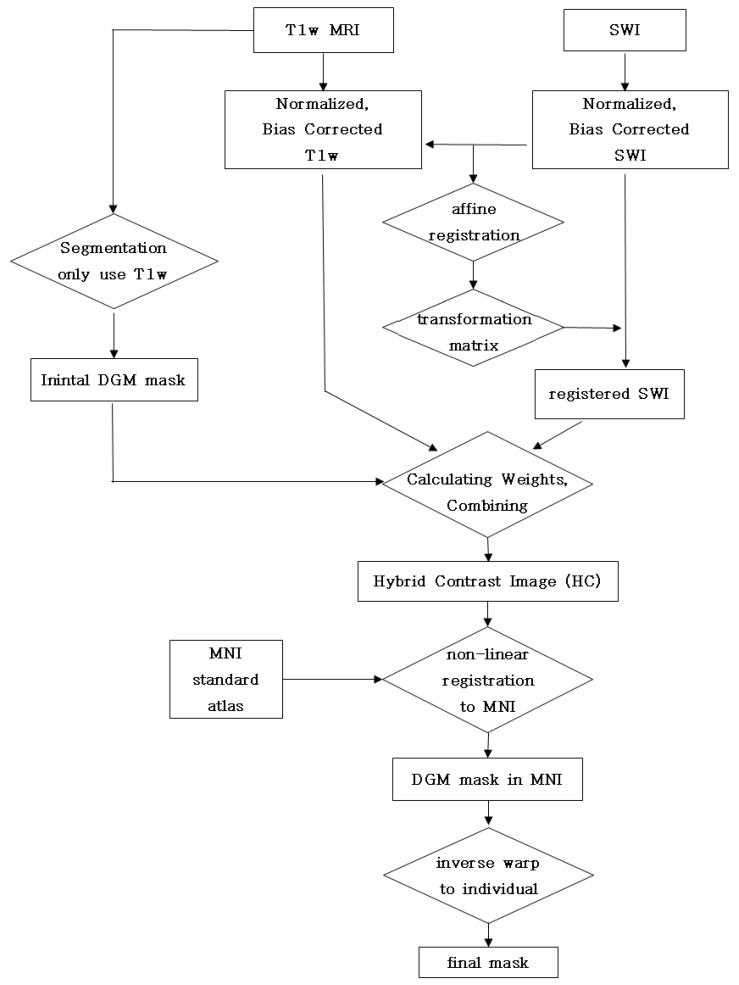
Flowchart of making a deep gray matter (DGM) mask using the T1w and SWI images. T1w and SWI were preprocessed through normalization, bias correction, and registration. The merging weight coefficients were calculated from initial DGM mask obtained using only T1w segmentation, and a hybrid contrast image (HC) was created as a result. The DGM mask was obtained by registering the HC to the MNI atlas space using non-linear registration. The final mask was obtained by applying inverse warping to the original coordinates.

Subsequently, the SWI images were registered to the T1w images using affine transform. Since the T1w and the SWI images of the same subject have identical anatomy and head motion between scans, the two images were successfully aligned using an affine registration. These data preprocessing are the steps before calculating weights, combining the steps shown in Figure 3.

### 2.4. SWI Segmentation Using Hybrid Contrast Image

To obtain segmentation results reflecting iron-related signals, we used both the T1w and SWI images simultaneously and merged them into a single hybrid contrast (HC) image [30]. Since SWI provides superior contrast for iron-rich structures, while the T1w images have greater contrast in the curvature of complicated gyrus and sulcus principally used for registration, using the HC results in the DGM segmentation that reflects more iron contents than using T1w alone, which better reflects the disease’s hallmarks such as nuclei atrophy [31] caused by the iron deposition.

The HC image is defined by linearly combining T1w and SWI images:

(1) $$\mathrm{HC}=w_{1}\cdot T1w+w_{2}\cdot \mathrm{SWI}\;,$$

where $w_{1}$ and $w_{2}$ are weighting coefficients for T1w and SWI, respectively.

We adjusted the weighting coefficients $w_{1}$ and $w_{2}$ to make HC as close as possible to the reference, Montreal Neurological Institute (MNI) template. We employed the MNI template’s contrast as the target for the coefficient optimization because it has a typical T1w contrast with outstanding DGM structural delineation. The optimized values of the weighting coefficients $w_{1}^{*}$, $w_{2}^{*}$ can be obtained by minimizing the squared difference of the mean signal intensities in the target brain regions between the HC and MNI template:

(2) $$\left(w_{1}^{*},w_{2}^{*}\right)=\underset{w_{1},w_{2}}{arg min}{∥\left[\begin{array}{cc} I_{\mathrm{put}}^{T1w} & I_{\mathrm{put}}^{\mathrm{SWI}}\\ I_{\mathrm{pall}}^{T1w} & I_{\mathrm{pall}}^{\mathrm{SWI}} \end{array}\right]\left[\begin{array}{c} w_{1}\\ w_{2} \end{array}\right]-\left[\begin{array}{c} I_{\mathrm{put}}^{\mathrm{MNI}}\\ I_{\mathrm{pall}}^{\mathrm{MNI}} \end{array}\right]∥}_{2}^{2}\;,$$

where $I_{\mathrm{put}}^{T1w}$, $I_{\mathrm{put}}^{\mathrm{SWI}}$, and $I_{\mathrm{put}}^{\mathrm{MNI}}$ are the mean values of the T1w, SWI, and MNI template images in the putamen region, respectively, and $I_{\mathrm{pall}}^{T1w}$, $I_{\mathrm{pall}}^{\mathrm{SWI}}$, and $I_{\mathrm{pall}}^{\mathrm{MNI}}$ are the mean values of the T1w, SWI, and MNI template images in the globus pallidus region, respectively. We chose the putamen and globus pallidus for the target regions because of high-contrast signals in the broad areas.

Then, we used advanced normalization tools (ANTs) to register the HC to the MNI template by computing an initial affine registration and non-linear registration employing a non-rigid diffeomorphic registration scheme [32]. The ANTs produced the most consistent and reliable registration results among 14 different registration methods [33]. The segmentation results from the MNI space were inversely warped to the individual T1w image space. The overall procedure of SWI segmentation is shown in Figure 3.

### 2.5. Feature Extraction and Selection

Radiomic features were extracted from the segmented DGM region of the SWI images automatically computed in Section 2.4. The radiomic features included 19 first-order statistical features, 10 2D shape-based features, 16 3D shape-based features and the following texture-based features: 72 gray-level co-occurrence matrix (GLCM) features, 16 gray-level run length matrix (GLRLM) features, 16 gray-level size zone matrix (GLSZM) features, 15 neighboring gray-tone difference matrix (NGTDM) features, and 14 gray-level dependence matrix (GLDM) features [34], as shown in Figure 2. These matrices represent the relationship with the surrounding voxels according to the kernel for each voxel. For example, the (i,j) th element of the GLCM represents the number of times the combination of levels *i* and *j* occur in two voxels in the image, which are separated by a distance of δ pixels along the angle θ.

We added GLCM and NGTDM features while changing the distance to neighboring voxels for which the relationship was calculated as four and seven voxels to the default python radiomic package [35]. Since SWI does not provide quantitative measurements of susceptibility, we excluded the signal-based features and focused on the texture-based features. We subtracted the signal-based features such as the minimum, maximum, mean, median, 10th percentile, 90th percentile of intensity, gray level range, and others. We use only selected optimal features (see below).

Among the sub-cortical structures in the DGM, we chose the putamen to extract radiomic features because comparing them is easy owing to the putamen’s large size and high contrast. It shows a large difference between the mask segmented from the T1w-only and the proposed methods. In addition, the radiomic results extracted from the putamen showed the best performance in disease classification using machine learning [8].

Next, to avoid overfitting the learning model, feature selection was performed before applying machine learning algorithms [36]. We employed the Fisher score algorithm to rank the radiomic features and a filter-based method for supervised feature selection. It chooses each feature independently according to its scores based on the Fisher criterion. We selected the top-10 ranked features based on Fisher score. We finally applied these selected features to classify the data using machine learning.

### 2.6. Machine Learning Classifier Training and Testing

To distinguish between subtypes of parkinsonian syndromes, we used the 10 most popular machine learning classifiers [37] such as k-nearest neighbors (kNN) [38], linear support vector machine classifier (linSVC) [39], support vector machine with radial basis function (RBF) kernel classifier (RBFSVC) [40], Gaussian process classifier (GP) [41], random forest classifier (RF) [42], decision tree classifier (DT) [43], multi-layer perceptron classifier (MLP) [44], AdaBoost classifier (ADA) [45], Gaussian Naïve Bayes classifier (GNB) [46], and quadratic discriminant analysis classifier (QDA) [47]. These classifiers have the potential for radiomics to aid in the development of non-invasive biomarkers [48].

The total datasets were divided into training and testing sets at a 7:3 ratio. In the training sets, features were selected, and 10 classifiers were constructed with 3-fold cross-validation. To evaluate the performance of the classifiers for differentiation of APS, the area under receiver operating characteristic curve (AUC), balanced accuracy (bAcc), sensitivity (Sen), specificity (Spe), and accuracy (Acc) were measured as defined by:

(3) $$\mathrm{Sen}=\frac{\mathrm{TP}}{\mathrm{TP}+\mathrm{FN}}$$

(4) $$\mathrm{Spe}=\frac{\mathrm{TN}}{\mathrm{TN}+\mathrm{FP}}$$

(5) $$\mathrm{bAcc}=\frac{\mathrm{Sen}+\mathrm{Spe}}{2}$$

(6) $$\mathrm{Acc}=\frac{\mathrm{TP}+\mathrm{TN}}{\mathrm{TP}+\mathrm{TN}+\mathrm{FP}+\mathrm{FN}}$$

where TP denotes the number of the actual positives that are correctly classified as positives, FN denotes the number of the actual positives that are wrongly classified as negatives, TN denotes the number of the actual negatives that are correctly classified as negatives, and FP denotes the number of the actual negatives that are wrongly classified as positives. The AUC metric is defined as the area under the receiver operating characteristic (ROC) curve plotted by true positive rate (TPR, equivalent to sensitivity) against false positive rate (FPR, equivalent to 1−specificity) with varying thresholds. For statistical evaluation, the performance metrics were obtained by randomly changing the training and testing sets 100 times and averaged. The source code is available in GitHub: https://github.com/KimYunSoo/classify_radiomic (accessed on 22 January 2022).

## 3. Results

### 3.1. Demographic Characteristics

The demographic and clinical characteristics of the subject groups are listed in Table 2. There were no significant differences between subject groups in terms of gender distribution. Age was higher in the PSP group than other groups. There was no discernible difference in disease duration between MSA-P, MSA-C, PSP, and PD. The disease severity measured using the UPDRS and H&Y scores was greater in the PSP and MSA groups than the PD group, and MMSE was lower in the PSP and MSA groups than the PD group (*p* < 0.001).

### 3.2. SWI Segmentation Results

**Figure 4:**
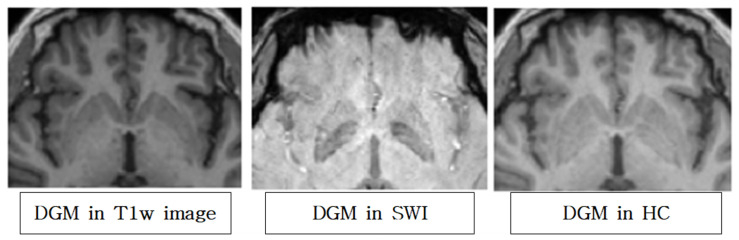
Deep gray matter (DGM) axial slice in T1w, SWI, and HC images. HC has both a high contrast cortex, which is the advantage of T1w, and a more prominent DGM boundary, which is visible in the SWI.

**Figure 5:**
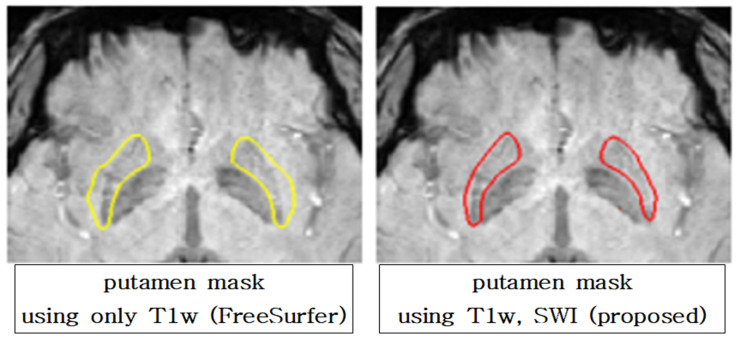
Putamen mask of segmentation result of using only T1-weighted image (FreeSurfer) and using T1w and SWI (proposed method) with SWI overlaid. The segmentation result using only T1w includes the part without iron accumulation when overlaid with the SWI (yellow). The proposed method reflects more of the iron deposition (red).

Figure 4 shows an example of axial slices around the DGM area in the T1w, SWI, and HC images. The DGM contrast is weak and the cortex contrast is clear in the T1w, while the trend is opposite for SWI. Whereas, the HC shows high contrast clearly for both the DGM and cortex. Figure 5 shows that the proposed approach produces segmentation results that better represent hypointensity indicating iron concentration in putamen SWI images. HC segmentation masks that use both T1w and SWI simultaneously reflect more hallmarks of parkinsonian disorders, such as iron accumulation and the resulting putamen atrophy, than T1w-only masks.

### 3.3. Feature Extraction and Selection Results

Table 3 shows the 10 most significant features selected from SWI for the differentiation of MSA-P and PD when using HC and T1w-only (by FreeSurfer, FS) segmentation masks and their mean values. Autocorrelation7, SumAverage4, JointAverage4, SumAverage7, JointAverage7, and Imc24 in GLCM and HighGrayLevelEmphasis in GLDM were commonly selected both in HC and T1w-only segmentation. Imc24 is the correlation between the probability distribution of intensity and occurrence number, quantifying the complexity of the texture, by neighboring voxel distances of 4. JointAverage7 and SumAverage7 (JointAverage4 and SumAverage4) measure the relationship between occurrences of pairs by neighboring voxel distances of 7 (4, respectively) with lower or higher intensity values. These indicate that the number of pairs of lower or higher intensities helps to differentiate between diseases. HighGrayLevelEmphasis in GLDM measures the distribution of the higher gray-level values with a higher value indicating a greater concentration of high gray-level values in the volume. In addition, in the case of comparison with other disease groups as shown in Table A1, Table A2, Table A3, Table A4 and Table A5, ClusterShade4 and MCC4 were also found to be common in HC and T1w-only. ClusterShade4 is a metric of the skewness and uniformity of the GLCM by neighboring voxel distances of 4 [49]. MCC4 is the maximal correlation coefficient for nearby voxel distances of 4, which also assesses the complexity of the texture. These features represent how dependent and uniform the distributions are.

The significant features only selected using HC mask include Autocorrelation4 in GLCM and HighGrayLevelRunEmphasis in GLRLM. Autocorrelation4 quantifies the magnitude of texture coarseness by neighboring voxel distances of 4; therefore, it operates more effectively in the HC segmentation mask as clusters of similar intensities appear better in HC than in T1w-only mask, which includes regions that are not iron-deposited. HighGrayLevelRunEmphasis in GLRLM measures the distribution of the higher gray-level values. RunEntropy and ShortRunHighGrayLevelEmphasis in GLRLM are also common when using HC masks in other disease group comparisons. RunEntropy is a metric that evaluates the uncertainty and randomness in the distribution of run lengths and gray levels. Therefore, heterogeneity in the texture patterns measure by RunEntropy is helpful in classifying each disorder. ShortRunHighGrayLevelEmphasis assesses the distribution of the high gray-level values and their joint distribution with shorter run lengths in GLRLM. The feature indicates how concentrated hyperintensities in SWI are, which is significant for distinguishing each subtype of parkinsonian disorder.

The significant features selected using T1w-only segmentation include GrayLevelNonUniformity in GLRLM and DependenceVariance in GLDM. GrayLevelNonUniformity is a metric that compares the similarity of the SWI image’s gray-level intensity values. The variance in dependence size in the image is measured by DependenceVariance. Moreover, in other disorder comparison cases, LargeDependenceHighGrayLevelEmphasis in the GLDM and Strength in NGTDM were frequently selected features using the T1w-only mask. LargeDependenceHighGrayLevelEmphasis in GLDM is the metric of joint distribution of substantial reliance on it. Strength in NGTDM measures how easily defined and visible the primitives in the image are. These all work mainly in the T1w-only mask, where there are both hypo- and hyper-intensity clusters together, because the T1w-only mask is likely to include the region without iron deposition (see Figure 5).

### 3.4. SVM Results

Table 4 lists the training and testing area under the receiver operating characteristic curve (AUC) of the RBF SVM classifier employing features from the T1w-only and HC masks. The SVM with RBF kernel that learns the radiomic features extracted from iron-reflected segmentation results produced an average AUC of 0.8607 in training and 0.8489 in testing. T1w-only mask-based radiomic training classifiers had an average AUC of 0.7570 in training and 0.7866 in testing. The classifier model trained with features extracted using the HC mask shows better performance than the T1w-only mask-based SVM classifier.

**Figure A1:**
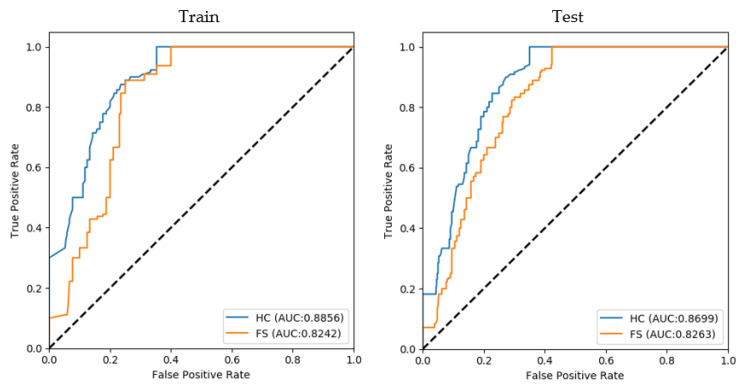
Receiver operating characteristic (ROC) curves of the RBF SVM classifier for MSA-P vs. MSA-C.

**Figure A2:**
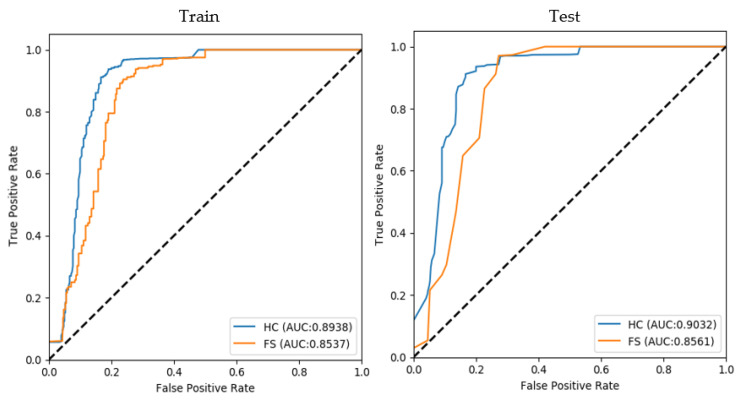
Receiver operating characteristic (ROC) curves of the RBF SVM classifier for MSA-P vs. PD.

**Figure A3:**
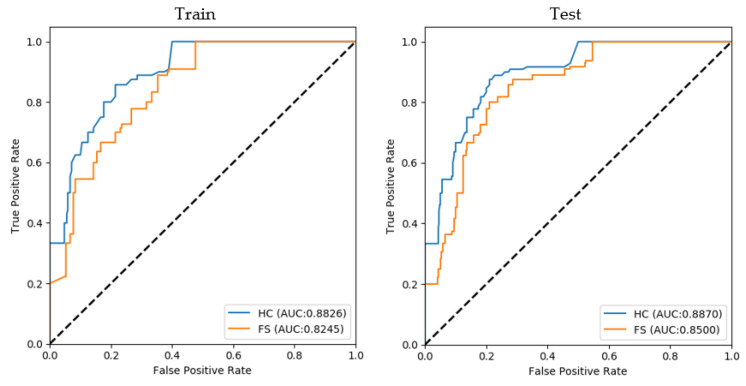
Receiver-operating characteristic (ROC) curves of the RBF SVM classifier for MSA-P vs. PSP.

**Figure A4:**
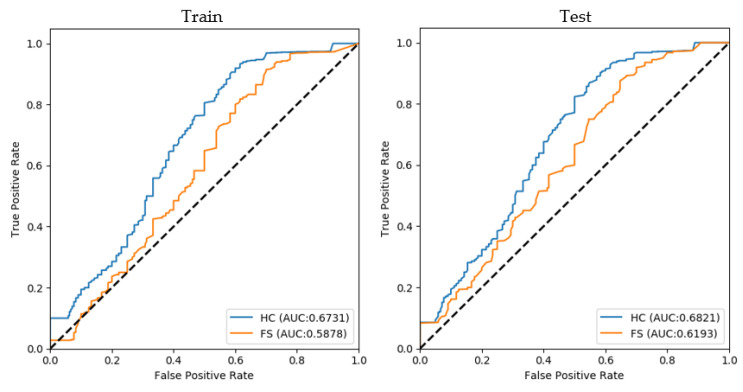
Receiver operating characteristic (ROC) curves of the RBF SVM classifier for MSA-C vs. PD.

**Figure A5:**
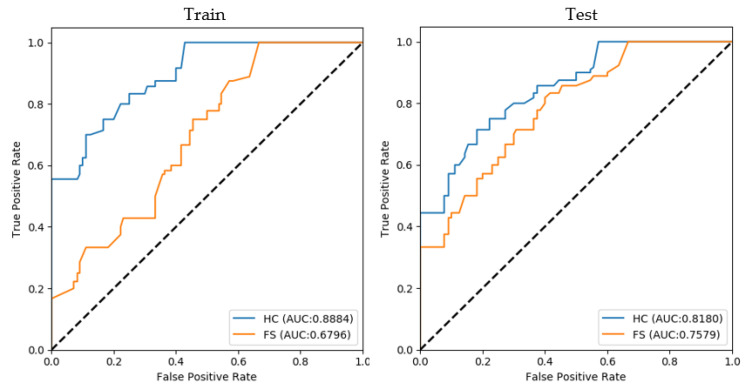
Receiver operating characteristic (ROC) curves of the RBF SVM classifier for MSA-C vs. PSP.

**Figure A6:**
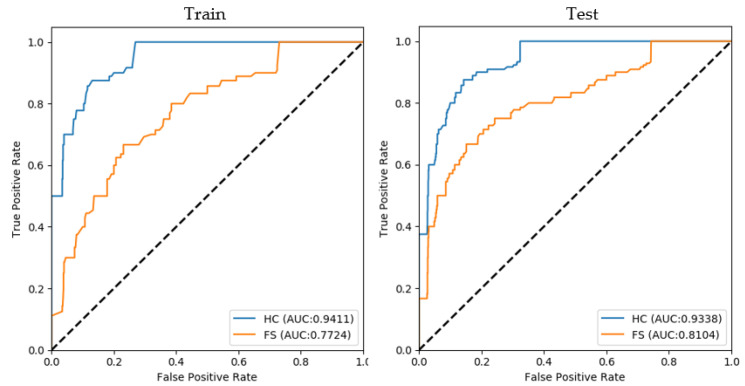
Receiver operating characteristic (ROC) curves of the RBF SVM classifier for PD vs. PSP.

The RBF SVM classifier receiver operating characteristic (ROC) curves for each disease distinguishing case are shown in Figure A1, Figure A2, Figure A3, Figure A4, Figure A5 and Figure A6. Through other classification algorithms, it was confirmed that the performance of the proposed method is improved compared to the T1w-only method in the same way as the RBF SVM.

The balanced accuracy, sensitivity, and specificity of the RBF SVM classifier using features from T1w-only masks and HC masks are listed in Table 5. The machine learning classifier that learns the SWI-reflected radiomic features produced an average balanced accuracy of 0.7666 for the training cohort and 0.7992 for the testing cohort. The classifier model trained by radiomics extracted from T1w-only segmentation masks achieved 0.6557 in training and 0.7620 in testing.

The classifier that was trained on the radiomic features extracted by the proposed method achieved an average accuracy of 0.8000 in training and 0.8059 in testing, as shown in Table 6. Conventional T1w-only segmentation classifiers had an accuracy of 0.7352 in training and 0.7653 in testing.

The AUC, balanced accuracy, sensitivity, specificity, and accuracy of all other classifiers are listed in Table A6, Table A7, Table A8, Table A9, Table A10, Table A11, Table A12, Table A13, Table A14, Table A15, Table A16, Table A17, Table A18, Table A19, Table A20, Table A21, Table A22, Table A23, Table A24, Table A25, Table A26, Table A27, Table A28, Table A29, Table A30, Table A31 and Table A32. Similar to the RBF SVM, in other classifier models, the AUC, balanced accuracy, and accuracy increased when HC masks reflecting iron-related signal were used.

## 4. Discussion and Conclusions

In this paper, we proposed a novel framework that automatically analyzes the disease-specific patterns of iron deposition using SWI. Through this proposed framework, by directly inputting raw data, the results of disease classification by automated processing without any human intervention can be applied to diagnosis.

Atypical Parkinsonian syndromes, such as MSA-P, MSA-C, and PSP, can be mistaken for PD, especially in the early stages of the disease. This is because both APS and PD are present with Parkinsonism. Therefore, it is critical to distinguish between PD and APS; nevertheless, conventional MRI still makes it difficult to discriminate between these neurodegenerative disorders.

We demonstrated that in individuals with abnormal brain anatomy, the commonly used T1w-only segmentation pipeline produces erroneous subcortical segmentation. The goal of this study was to overcome this issue by modifying the conventional pipeline that incorporates nonlinear registration and by using a dedicated hybrid image contrast created by combining standard T1w images with SWI. By using the HC, which is a combination of the T1w and SWI, for the DGM segmentation, it is possible to identify iron deposition automatically without manual segmentation by expert radiologists, as was done in the past. We have visually shown that putamen segmentation performance was improved by using both the T1w and SWI.

We conducted a qualitative assessment of the visual delineation of our segmentation framework results. If there is a manual segmentation mask by an expert, it can be used as the gold standard, and objective and quantitative evaluation can be performed through metrics such as the dice coefficient. However, manual segmentation performed by experts is costly and time consuming. Some studies have used visual ratings as metrics [50].

Another goal of the present study was to a create machine learning classifier that can distinguish APS from PD using image texture-based features derived from basal nuclei on SWI. Different iron deposition patterns for each disease were compared by extracting quantified radiomic features. The distinction between each subtype of parkinsonian disorder groups was better exposed by the features retrieved with the SWI-reflected mask. When classifying diseases using various machine learning algorithms, it was confirmed that the performance of the classifier improved by training features extracted from the HC.

We recognize the lack of pathological confirmation for diagnosis and phenotypic categorization, which remain the gold standard for the diagnosis of PSP. However, we selected patients with the typical clinical characteristics of MSA, PSP and PD, and assessed these patients over several years.

We used the texture features of the signal intensity contrast to train the machine learning classifiers. Since SWI does not represent a quantified value of iron content, the quantitative values of iron deposition were not measured. We used only texture features because we intended to classify disorders by analyzing the image patterns of each disease and not to create a reference point or threshold with a quantified number. Although we did not directly compare the quantitative values, we indirectly demonstrated the improvement of segmentation through outperforming the machine learning classifier.

In future work, we will validate the proposed framework more clinically using R2*. In addition, we will aim to apply our hybrid approach of brain tissue segmentation in other PET-MRI modalities.

## Figures and Tables

**Table 1:** An overview of the common neuroimaging modalities (DTI, PET, SPECT, and SWI), role of modality, and potential of differentiating PD and APS.

Neuroimaging Modality	Role of Modality	Potential of Differentiating PD and APS
Diffusion-tensor image (DTI) [7]	Detect characteristics such as fractional anisotropy (FA) and mean diffusion (MD)	Decreased FA and/or increased MD in the substantia nigra, the corpus callosum, the frontal lobes, the cingulum, and the temporal cortex
Positron emission tomography (PET) [9]	Measure amyloid pathology, tau pathology, a-Synuclein pathology, metabolic activity by measuring changes in the glucose consumption	PD-related spatial covariance pattern may involve increased pallidothalamic and pontine activity associated with decreased metabolism in supplementary motor area, premotor cortex, and parietal association areas
Single photon emission computed tomography (SPECT) [12]	Measure dopamine transporter (DAT) density, dopamine D2 receptor, metabolic activity by measuring changes in the cerebral blood flow	Decreased striatal presynaptic DAT binding contralateral to parkinsonian symptomatology with greater reduction in posterior putamen than in anterior putamen or caudate nucleus
Susceptibility weighted image (SWI) [13]	Visualize iron-related contents sensitively	Substantia nigra pars compacta, globus pallidus internus, the putamen, and the red nucleus have been described as regions with increased iron concentration

**Table 2:** Clinical and demographic characteristics of patients.

	MSA-P	MSA-C	PSP	PD	Significance
Gender (M/F)	13/21	9/12	11/6	32/24	*p* = 0.179 $\chi^{2}$(3) = 4.894
Age (years)	59.05 ± 7.83	58.95 ± 6.30	65.64 ± 5.58	56.85 ± 7.60	*p* < 0.001 F(3,124) = 6.052
UPDRS-III	39.73 ± 12.86	30.80 ± 9.73	35.94 ± 8.15	24.33 ± 9.57	*p* < 0.001 F(3,124) = 16.597
H-Y stage	3.10 ± 0.76	3.14 ± 0.61	3.5 ± 0.75	2.02 ± 0.51	*p* < 0.001 F(3,124) = 36.885
Duration (months)	30.23 ± 15.25	30.52 ± 13.62	31.11 ± 18.09	35.41 ± 22.23	*p* = 0.661 F(3,124) = 0.532
MMSE	25.44 ± 2.73	24.76 ± 3.23	23.82 ± 4.03	26.89 ± 2.41	*p* < 0.001 F(3,124) = 6.395

The data are presented as number or mean ± standard deviation. For continuous variables, values are expressed as F statistics, while for categorical variables, values are expressed as $\chi^{2}$ statistics. MSA-P : parkinsonian variant of multiple system atrophy, MSA-C: cerebellar variant of multiple system atrophy, PSP: progressive supranuclear palsy, PD: Parkinson’s disease, UPDRS III: motor examination part of the Unified Parkinson’s Disease Rating Scale, H-Y: Hoehn & Yahr.

**Table 3:** Mean values of top 10 features selected from SWI when comparing MSA-P and PD using HC and T1w-only segmentation masks. Common features found both in HC and T1w-only segmentation are indicated in bold.

Features by HC	MSA-P	PD	Features by T1w-Only	MSA-P	PD
glrlm_ShortRunHigh- GrayLevelEmphasis	51.2291	20.3952	glcm_MCC4	0.4166	0.2744
**glcm_Autocorrelation7**	69.2407	27.9998	**glcm_Imc24**	0.5665	0.364
**glcm_JointAverage7**	8.1353	5.1994	**glcm_JointAverage7**	8.4094	5.5243
**glcm_SumAverage7**	16.2706	10.3988	**glcm_SumAverage7**	16.8187	11.0487
**gldm_HighGrayLevelEmphasis**	64.9219	27.8777	gldm_DependenceVariance	23.2205	27.8954
glrlm_HighGrayLevelRunEmphasis	64.0086	28.202	glrlm_GrayLevelNonUniformity	344.9109	652.5307
**glcm_Imc24**	0.5442	0.3244	**glcm_Autocorrelation7**	72.6811	31.5304
glcm_Autocorrelation4	63.6349	26.3972	**glcm_SumAverage4**	16.1899	10.7769
**glcm_SumAverage4**	15.5239	10.0885	**glcm_JointAverage4**	8.095	5.3884
**glcm_JointAverage4**	7.762	5.0443	**gldm_HighGrayLevelEmphasis**	75.7005	32.9521

**Table 4:** RBF SVM classifier training and testing AUC when using HC and T1w-only segmentation masks. The classifier model trained with features extracted using HC masks showed 0.1037 higher AUC for training and 0.062 higher AUC for testing compared to the T1w-only mask-based SVM classifier.

Differentiating Diseases	Train AUC		Test AUC	
	HC	T1w-Only	HC	T1w-Only
MSA-P vs. MSA-C	0.8856	0.8242	0.8699	0.8263
MSA-P vs. PD	0.8938	0.8537	0.9032	0.8561
MSA-P vs. PSP	0.8825	0.8245	0.8869	0.8499
MSA-C vs. PD	0.6731	0.5878	0.6820	0.6193
MSA-C vs. PSP	0.8883	0.6796	0.8180	0.7578
PD vs. PSP	0.9411	0.7724	0.9338	0.8104

AUC: area under the receiver operating characteristic (ROC) curve

**Table 5:** RBF SVM classifier training and testing balanced accuracy, sensitivity, and specificity when using HC and T1w-only segmentation masks. The classifier model trained with features extracted using HC masks outperformed the SVM classifier based on T1w-only masks by 0.1109 in training and 0.0372 in testing in terms of the balanced accuracy.

Differentiating Diseases	Train						Test					
	HC			T1w-Only			HC			T1w-Only		
	bAcc	Sen	Spe	bAcc	Sen	Spe	bAcc	Sen	Spe	bAcc	Sen	Spe
MSA-P vs. MSA-C	0.7931	0.8472	0.7390	0.7005	0.8045	0.5963	0.7922	0.8662	0.7183	0.7313	0.8298	0.6327
MSA-P vs. PD	0.9120	0.8865	0.8937	0.8482	0.8316	0.8647	0.8981	0.9046	0.8917	0.8800	0.8958	0.8642
MSA-P vs. PSP	0.7790	0.8707	0.6874	0.6023	0.7854	0.4193	0.7862	0.8802	0.6922	0.7535	0.8345	0.6725
MSA-C vs. PD	0.7863	0.7727	0.7999	0.7516	0.7335	0.7698	0.7899	0.7988	0.7810	0.7872	0.8031	0.7714
MSA-C vs. PSP	0.7470	0.8045	0.6895	0.5491	0.6714	0.4269	0.7262	0.8020	0.6505	0.6828	0.6838	0.6818
PD vs. PSP	0.5823	0.7914	0.3732	0.4826	0.7757	0.1894	0.8027	0.8194	0.7860	0.7376	0.7807	0.0776

bAcc : balanced accuracy, Sen: sensitivity, Spe: specificity.

**Table 6:** RBF SVM classifier training and testing accuracy when using HC and T1w-only segmentation masks. The HC trained classifier distinguishes disorders better than the T1w-only trained classifier by 0.0648 in training and 0.0406 in testing.

Differentiating Diseases	Train ACC		Test ACC	
	HC	T1w-Only	HC	T1w-Only
MSA-P vs. MSA-C	0.7972	0.7336	0.8018	0.7552
MSA-P vs. PD	0.8944	0.8544	0.8928	0.8571
MSA-P vs. PSP	0.8087	0.6902	0.8135	0.7692
MSA-C vs. PD	0.7960	0.7682	0.7804	0.7708
MSA-C vs. PSP	0.7172	0.5973	0.7288	0.6616
PD vs. PSP	0.7867	0.7676	0.8184	0.778

## Data Availability

The data presented in this study are available on request from the corresponding author. The data are not publicly available due to privacy.

## Appendix A

Figure A1, Figure A2, Figure A3, Figure A4, Figure A5 and Figure A6 show the receiver operating characteristic (ROC) curves for each differentiating disease case. As in the RBF SVM, in other algorithms, the classifiers that learned the features extracted using HC masks performed better overall in terms of the AUC, balanced accuracy, and accuracy compared to the models trained using the conventional T1w-only masks.

## Appendix B

### Appendix B.1

Table A1 list the mean values of the features with HC and T1w-only masks when comparing MSA-P and MSA-C.

**Table A1:** Mean values of the features of each subtype of disease.

HC Features	MSA-P	MSA-C	T1w-Only Features	MSA-P	MSA-C
glszm_GrayLevelVariance	6.8645	4.0758	glcm_Id4	0.4473	0.5428
glszm_HighGrayLevel- ZoneEmphasis	70.981	40.6072	glcm_ClusterTendency7	8.4945	3.201
ngtdm_Strength	0.2515	0.0898	ngtdm_Strength	0.2409	0.085
ngtdm_Strength4	0.1409	0.0534	gldm_SmallDependenceEmphasis	0.0638	0.0331
ngtdm_Strength7	0.1223	0.0495	gldm_SmallDependence- LowGrayLevelEmphasis	0.0021	0.0016
glrlm_ShortRunHigh- GrayLevelEmphasis	51.2291	24.4967	glcm_ClusterShade	−61.4694	−7.7083
glcm_Imc24	0.5442	0.3803	glcm_SumSquares4	6.3807	1.9578
glcm_JointAverage7	8.1353	5.728	glcm_Idm4	0.3788	0.4915
glcm_SumAverage7	16.2706	11.4561	glcm_ClusterShade4	−12.6393	−2.0909
glszm_SmallAreaHigh- GrayLevelEmphasis	34.8355	18.1394	gldm_DependenceVariance	23.2205	27.458

### Appendix B.2

Table A2 lists the mean values of the features with HC and T1w-only masks when comparing MSA-P and PSP.

**Table A2:** Mean values of the features of each subtype of disease.

HC Features	MSA-P	PD	T1w-Only Features	MSA-P	PD
glszm_ZoneVariance	10,415.4	16,002.22	ngtdm_Busyness4	6.2355	13.1834
ngtdm_Busyness	3.2868	6.393	glcm_DifferenceAverage7	2.6958	1.9655
glcm_JointAverage4	7.762	5.2133	gldm_GrayLevelNonUniformity	556.3621	736.7501
glcm_ClusterShade4	−11.6021	−0.0316	gldm_LargeDependence- HighGrayLevelEmphasis	7484.096	3824.813
gldm_SmallDependence- LowGrayLevelEmphasis	0.0027	0.0028	ngtdm_Strength	0.2409	0.1118
gldm_GrayLevelNonUniformity	438.2295	550.6763	gldm_HighGrayLevelEmphasis	75.7005	38.6685
gldm_DependenceNonUniformity	186.9766	175.5947	glcm_Idn4	0.8595	0.8683
glcm_Imc14	−0.074	−0.0451	glcm_DifferenceVariance4	4.8392	2.0783
glcm_MCC4	0.3823	0.2921	glrlm_GrayLevelNonUniformity	344.9109	470.024
glcm_Autocorrelation4	63.6349	28.8556	glszm_HighGrayLevel- ZoneEmphasis	75.1588	47.8979

### Appendix B.3

Table A3 lists the mean values of the features with HC and T1w-only masks when comparing MSA-C and PD.

**Table A3:** Mean values of the features of each subtype of disease.

HC Features	MSA-C	PD	T1w-Only Features	MSA-C	PD
glcm_ClusterShade	−7.7694	−1.3529	glcm_ClusterShade4	−2.0909	0.5222
glcm_ClusterShade4	−2.4839	−0.4058	glcm_ClusterShade	−7.7083	−0.0626
glcm_MCC4	0.2742	0.2319	glcm_MCC4	0.3167	0.2744
glcm_Imc14	−0.0384	−0.0291	glcm_JointAverage7	5.8946	5.5243
glcm_Imc24	0.3803	0.3244	glcm_ClusterShade7	−1.1194	−0.1304
glrlm_RunEntropy	3.8955	3.7793	gldm_DependenceVariance	27.458	27.8954
glcm_ClusterShade7	−1.1463	−0.3319	glcm_Imc24	0.422	0.364
gldm_DependenceEntropy	6.499	6.3396	glcm_Imc1	−0.2039	−0.1892
glrlm_GrayLevelNon- UniformityNormalized	0.2082	0.2397	gldm_DependenceEntropy	6.6475	6.518
glcm_SumEntropy	3.1802	2.9418	glcm_MCC	0.6602	0.6362

### Appendix B.4

Table A4 lists the mean values of the features with HC and T1w-only masks when comparing MSA-C and PSP.

**Table A4:** Mean values of the features of each subtype of disease.

HC Features	MSA-P	PD	T1w-Only Features	MSA-P	PD
glcm_MCC	0.6103	0.6006	gldm_DependenceVariance	27.458	21.3893
glrlm_RunEntropy	3.8955	3.8289	gldm_DependenceNon- UniformityNormalized	0.0555	0.0642
glcm_JointAverage7	5.728	5.4607	ngtdm_Coarseness	0.0024	0.0028
glcm_Imc24	0.3803	0.4196	glrlm_RunEntropy	4.0075	4.0395
glcm_MCC4	0.2742	0.2921	glszm_LargeAreaHigh- GrayLevelEmphasis	1,495,719	946,080.3
gldm_LargeDependence- LowGrayLevelEmphasis	6.085	6.0346	glszm_LargeAreaLow- GrayLevelEmphasis	1948.349	1398.383
gldm_DependenceNonUniformity	195.5685	175.5947	glszm_LowGrayLevelZoneEmphasis	0.0673	0.0777
glszm_ZoneVariance	31,931.73	16,002.22	gldm_LargeDependenceEmphasis	138.6942	109.827
glszm_SmallAreaLow- GrayLevelEmphasis	0.0275	0.0305	gldm_GrayLevelVariance	2.1446	2.9733
glszm_ZoneEntropy	5.0788	5.0619	gldm_SmallDependence- LowGrayLevelEmphasis	0.0016	0.002

### Appendix B.5

Table A5 lists the mean values of the features with HC and T1w-only masks when comparing PD and PSP.

**Table A5:** Mean values of the features of each subtype of disease.

HC Features	MSA-P	PD	T1w-Only Features	MSA-P	PD
glcm_Autocorrelation7	27.9998	31.5698	glcm_SumEntropy4	2.7913	3.1555
glcm_Contrast7	2.7699	4.5716	glcm_SumAverage7	11.0487	11.8343
gldm_LargeDependenceHigh- GrayLevelEmphasis	3796.027	2722.489	gldm_HighGrayLevelEmphasis	32.9521	38.6685
glrlm_RunEntropy	3.7793	3.8289	gldm_LargeDependence- HighGrayLevelEmphasis	4652.988	3824.813
glcm_DifferenceAverage4	1.1238	1.4629	gldm_LowGrayLevelEmphasis	0.0474	0.0488
gldm_DependenceVariance	27.5647	19.9317	glszm_ZonePercentage	0.0192	0.0266
glcm_ClusterProminence4	29.37	74.0809	glcm_JointEnergy4	0.0702	0.0402
glcm_JointAverage4	5.0443	5.2133	glcm_ClusterShade	-0.0626	0.2446
glcm_Imc24	0.3244	0.4196	glcm_DifferenceEntropy4	2.007	2.306
glrlm_RunPercentage	0.6247	0.6961	glszm_SizeZoneNonUniformity	17.0448	19.3692

## Appendix C

### Appendix C.1

Table A6, Table A7 and Table A8 list the results of the classifier trained with k-nearest neighbor (kNN).

**Table A6:** kNN classifier training and testing AUC when using HC and T1w-only segmentation masks.

Differentiating Diseases	Train		Test	
	HC	T1w-Only	HC	T1w-Only
MSA-P vs. MSA-C	0.8589	0.7677	0.8621	0.7910
MSA-P vs. PD	0.8839	0.8203	0.8865	0.8340
MSA-P vs. PSP	0.8357	0.8356	0.8569	0.8272
MSA-C vs. PD	0.6870	0.6613	0.6805	0.6613
MSA-C vs. PSP	0.7908	0.7855	0.7932	0.7813
PD vs. PSP	0.8895	0.7323	0.8761	0.8369

**Table A7:** kNN classifier training and testing balanced accuracy, sensitivity, and specificity using HC and T1w-only segmentation masks.

Differentiating Diseases	Train						Test					
	HC			T1w-Only			HC			T1w-Only		
	bAcc	Sen	Spe	bAcc	Sen	Spe	bAcc	Sen	Spe	bAcc	Sen	Spe
MSA-P vs. MSA-C	0.7915	0.8581	0.7250	0.6843	0.7889	0.5797	0.7906	0.8542	0.7269	0.7108	0.8054	0.6161
MSA-P vs. PD	0.8775	0.8682	0.8868	0.8057	0.7711	0.8402	0.8569	0.8259	0.8879	0.7924	0.7226	0.8622
MSA-P vs. PSP	0.7791	0.8574	0.7009	0.7712	0.8403	0.7020	0.8101	0.8803	0.7399	0.7867	0.8860	0.6874
MSA-C vs. PD	0.6513	0.4944	0.8082	0.6604	0.5292	0.7915	0.6779	0.5617	0.7949	0.6347	0.4776	0.7918
MSA-C vs. PSP	0.7196	0.7360	0.7032	0.6741	0.7237	0.6246	0.7242	0.7380	0.7103	0.6973	0.7305	0.6642
PD vs. PSP	0.8315	0.9033	0.7597	0.6147	0.8178	0.4116	0.8094	0.8981	0.7207	0.7330	0.8731	0.5928

bAcc: balanced accuracy, Sen: sensitivity, Spe: specificity.

**Table A8:** kNN classifier training and testing accuracy using HC and T1w-only segmentation masks.

Differentiating Diseases	Train		Test	
	HC	T1w-Only	HC	T1w-Only
MSA-P vs. MSA-C	0.7955	0.6970	0.8006	0.7333
MSA-P vs. PD	0.8789	0.8163	0.8642	0.8095
MSA-P vs. PSP	0.8055	0.7929	0.8277	0.8123
MSA-C vs. PD	0.7387	0.7379	0.7420	0.722
MSA-C vs. PSP	0.7070	0.6644	0.7167	0.7016
PD vs. PSP	0.8667	0.7324	0.8569	0.8116

### Appendix C.2

Comparison of the linear support vector machine (linSVM) classifier is given in Table A9, Table A10 and Table A11.

**Table A9:** linSVM classifier training and testing AUC when using HC and T1w-only segmentation masks.

Differentiating Diseases	Train		Test	
	HC	T1w-Only	HC	T1w-Only
MSA-P vs. MSA-C	0.8809	0.8790	0.8703	0.8631
MSA-P vs. PD	0.9159	0.8902	0.9156	0.8799
MSA-P vs. PSP	0.8840	0.8882	0.8928	0.8821
MSA-C vs. PD	0.7314	0.7097	0.7408	0.7261
MSA-C vs. PSP	0.9694	0.9349	0.9381	0.9300
PD vs. PSP	0.9433	0.8232	0.9346	0.8294

**Table A10:** linSVM classifier training and testing balanced accuracy, sensitivity, and specificity using HC and T1w-only segmentation masks.

Differentiating Diseases	Train						Test					
	HC			T1w-Only			HC			T1w-Only		
	bAcc	Sen	Spe	bAcc	Sen	Spe	bAcc	Sen	Spe	bAcc	Sen	Spe
MSA-P vs. MSA-C	0.7995	0.8693	0.7297	0.7540	0.8907	0.6173	0.7832	0.8562	0.7102	0.7700	0.8962	0.6437
MSA-P vs. PD	0.8998	0.9178	0.8818	0.8593	0.8524	0.8662	0.8964	0.9075	0.8854	0.8495	0.8383	0.8607
MSA-P vs. PSP	0.7720	0.8705	0.6735	0.7459	0.8376	0.6542	0.7904	0.8824	0.6983	0.7733	0.8834	0.6633
MSA-C vs. PD	0.7830	0.7737	0.7923	0.7899	0.8111	0.7687	0.8212	0.8753	0.7670	0.7854	0.8026	0.7682
MSA-C vs. PSP	0.9038	0.8987	0.9089	0.8210	0.8166	0.8254	0.8573	0.8552	0.8594	0.8500	0.8355	0.8645
PD vs. PSP	0.8291	0.9108	0.7475	0.6747	0.8329	0.5166	0.8314	0.9140	0.7489	0.7377	0.8481	0.6272

bAcc: balanced accuracy, Sen: sensitivity, Spe: specificity.

**Table A11:** linSVM classifier training and testing accuracy using HC and T1w-only segmentation masks.

Differentiating Diseases	Train		Test	
	HC	T1w-Only	HC	T1w-Only
MSA-P vs. MSA-C	0.8052	0.7890	0.7921	0.7738
MSA-P vs. PD	0.8926	0.8619	0.8892	0.8392
MSA-P vs. PSP	0.8002	0.7732	0.8161	0.8019
MSA-C vs. PD	0.7883	0.7697	0.7691	0.7670
MSA-C vs. PSP	0.8907	0.8125	0.8419	0.8340
PD vs. PSP	0.8638	0.7781	0.8742	0.8127

### Appendix C.3

Table A12, Table A13 and Table A14 list the results of the Gaussian process (GP) based classifier.

**Table A12:** GP classifier training and testing AUC using HC and T1w-only segmentation masks.

Differentiating Diseases	Train		Test	
	HC	T1w-Only	HC	T1w-Only
MSA-P vs. MSA-C	0.7420	0.6935	0.8529	0.6632
MSA-P vs. PD	0.9243	0.8753	0.9141	0.8790
MSA-P vs. PSP	0.8880	0.8277	0.8936	0.8735
MSA-C vs. PD	0.7018	0.6893	0.7185	0.6957
MSA-C vs. PSP	0.7354	0.7274	0.7574	0.7322
PD vs. PSP	0.6305	0.5029	0.5221	0.5000

**Table A13:** GP classifier training and testing balanced accuracy, sensitivity, and specificity when using HC and T1w-only segmentation masks.

Differentiating Diseases	Train						Test					
	HC			T1w-Only			HC			T1w-Only		
	bAcc	Sen	Spe	bAcc	Sen	Spe	bAcc	Sen	Spe	bAcc	Sen	Spe
MSA-P vs. MSA-C	0.7357	0.7826	0.6887	0.6948	0.7990	0.5905	0.7868	0.8738	0.6998	0.7388	0.8015	0.6762
MSA-P vs. PD	0.8916	0.8982	0.8849	0.8449	0.8238	0.8661	0.9024	0.9097	0.8951	0.8800	0.8958	0.8642
MSA-P vs. PSP	0.7412	0.8423	0.6401	0.7171	0.8159	0.6182	0.7590	0.8596	0.6584	0.7393	0.8532	0.6253
MSA-C vs. PD	0.7858	0.7922	0.7794	0.7781	0.7555	0.8006	0.7993	0.818	0.7806	0.7824	0.7836	0.7812
MSA-C vs. PSP	0.6655	0.7110	0.6201	0.6098	0.5594	0.6603	0.7171	0.7104	0.7238	0.6076	0.5661	0.6491
PD vs. PSP	0.7260	0.7909	0.6611	0.5803	0.8032	0.3574	0.7718	0.8081	0.7355	0.7699	0.7990	0.7408

bAcc: balanced accuracy, Sen: sensitivity, Spe: specificity.

**Table A14:** GP classifier training and testing accuracy when using HC and T1w-only segmentation masks.

Differentiating Diseases	Train		Test	
	HC	T1w-Only	HC	T1w-Only
MSA-P vs. MSA-C	0.7515	0.7369	0.7933	0.7682
MSA-P vs. PD	0.8901	0.8530	0.8964	0.8571
MSA-P vs. PSP	0.7918	0.7690	0.8032	0.7884
MSA-C vs. PD	0.7929	0.78	0.7816	0.7770
MSA-C vs. PSP	0.6720	0.5594	0.7096	0.5661
PD vs. PSP	0.7857	0.7505	0.8033	0.8009

### Appendix C.4

Table A15, Table A16 and Table A17 list the performances of the classifier that learned radiomic features based on random forest (RF).

**Table A15:** RF classifier training and testing AUC using HC and T1w-only segmentation masks.

Differentiating Diseases	Train		Test	
	HC	T1w-Only	HC	T1w-Only
MSA-P vs. MSA-C	0.8725	0.7894	0.8840	0.8504
MSA-P vs. PD	0.9135	0.8400	0.9078	0.8553
MSA-P vs. PSP	0.8462	0.8418	0.8632	0.8497
MSA-C vs. PD	0.7159	0.6951	0.7099	0.6649
MSA-C vs. PSP	0.9641	0.8880	0.9458	0.8152
PD vs. PSP	0.8896	0.8260	0.8869	0.8648

**Table A16:** RF classifier training and testing balanced accuracy, sensitivity, and specificity using HC and T1w-only segmentation masks.

Differentiating Diseases	Train						Test					
	HC			T1w-Only			HC			T1w-Only		
	bAcc	Sen	Spe	bAcc	Sen	Spe	bAcc	Sen	Spe	bAcc	Sen	Spe
MSA-P vs. MSA-C	0.8168	0.8737	0.7599	0.7366	0.8170	0.6561	0.8138	0.8863	0.7412	0.7826	0.8847	0.6804
MSA-P vs. PD	0.8879	0.8924	0.8833	0.8301	0.8088	0.8514	0.8924	0.9013	0.8835	0.8645	0.8573	0.8717
MSA-P vs. PSP	0.7734	0.8299	0.7169	0.7262	0.8144	0.6380	0.7781	0.8530	0.7032	0.7753	0.8529	0.6976
MSA-C vs. PD	0.7196	0.6327	0.8065	0.7101	0.6278	0.7924	0.7359	0.6821	0.7898	0.7192	0.6453	0.7932
MSA-C vs. PSP	0.8920	0.8921	0.8918	0.7960	0.8117	0.7803	0.8674	0.8612	0.8736	0.8192	0.8233	0.8152
PD vs. PSP	0.7798	0.8696	0.6901	0.6968	0.8322	0.5614	0.7822	0.8772	0.6872	0.7234	0.8523	0.5945

bAcc: balanced accuracy, Sen: sensitivity, Spe: specificity.

**Table A17:** RF classifier training and testing accuracy using HC and T1w-only segmentation masks.

Differentiating Diseases	Train		Test	
	HC	T1w-Only	HC	T1w-Only
MSA-P vs. MSA-C	0.826	0.7475	0.8212	0.7902
MSA-P vs. PD	0.8855	0.8363	0.8857	0.8571
MSA-P vs. PSP	0.7941	0.7643	0.7994	0.7987
MSA-C vs. PD	0.7762	0.7639	0.7691	0.7670
MSA-C vs. PSP	0.8884	0.7839	0.8517	0.8083
PD vs. PSP	0.8295	0.7762	0.8336	0.8051

### Appendix C.5

Decision tree (DT) classifier results are listed in Table A18, Table A19 and Table A20.

**Table A18:** DT classifier training and testing AUC using HC and T1w-only segmentation masks.

Differentiating Diseases	Train		Test	
	HC	T1w-Only	HC	T1w-Only
MSA-P vs. MSA-C	0.7789	0.6615	0.7528	0.7000
MSA-P vs. PD	0.8568	0.7556	0.8688	0.7521
MSA-P vs. PSP	0.7426	0.7495	0.7631	0.7577
MSA-C vs. PD	0.6427	0.6192	0.6465	0.6208
MSA-C vs. PSP	0.8734	0.7890	0.8412	0.8180
PD vs. PSP	0.7453	0.6802	0.7433	0.7119

**Table A19:** DT classifier training and testing balanced accuracy, sensitivity, and specificity using HC and T1w-only segmentation masks.

Differentiating Diseases	Train						Test					
	HC			T1w-Only			HC			T1w-Only		
	bAcc	Sen	Spe	bAcc	Sen	Spe	bAcc	Sen	Spe	bAcc	Sen	Spe
MSA-P vs. MSA-C	0.7650	0.8261	0.7040	0.6463	0.7504	0.5423	0.7409	0.8213	0.6605	0.6976	0.7371	0.6581
MSA-P vs. PD	0.8510	0.8383	0.8637	0.7745	0.7153	0.8337	0.8439	0.8189	0.8689	0.7752	0.6838	0.8666
MSA-P vs. PSP	0.7487	0.8268	0.6706	0.7375	0.8244	0.6507	0.7317	0.8353	0.6280	0.7271	0.8210	0.6332
MSA-C vs. PD	0.6842	0.5587	0.8096	0.6366	0.4693	0.8039	0.6529	0.5105	0.7953	0.6483	0.5017	0.7948
MSA-C vs. PSP	0.8725	0.8911	0.8539	0.7911	0.8082	0.7741	0.8438	0.8618	0.8257	0.8316	0.8295	0.8336
PD vs. PSP	0.7743	0.8768	0.6718	0.6794	0.8506	0.5082	0.7390	0.8808	0.5971	0.6934	0.8588	0.5281

bAcc: balanced accuracy, Sen: sensitivity, Spe: specificity.

**Table A20:** DT classifier training and testing accuracy using HC and T1w-only segmentation masks.

Differentiating Diseases	Train		Test	
	HC	T1w-Only	HC	T1w-Only
MSA-P vs. MSA-C	0.7712	0.6859	0.7539	0.7132
MSA-P vs. PD	0.8528	0.7888	0.8517	0.8035
MSA-P vs. PSP	0.7714	0.7746	0.7574	0.7561
MSA-C vs. PD	0.7497	0.7247	0.7329	0.7141
MSA-C vs. PSP	0.8712	0.7757	0.8378	0.8267
PD vs. PSP	0.8295	0.7581	0.8116	0.7878

### Appendix C.6

The performances of the classifier trained with multi-layer perceptron (MLP), also known as Neural Net (NN), are listed in Table A21, Table A22 and Table A23.

**Table A21:** MLP classifier training and testing AUC using HC and T1w-only segmentation masks.

Differentiating Diseases	Train		Test	
	HC	T1w-Only	HC	T1w-Only
MSA-P vs. MSA-C	0.8750	0.7818	0.8768	0.8620
MSA-P vs. PD	0.9029	0.8714	0.9157	0.8770
MSA-P vs. PSP	0.8924	0.8753	0.9072	0.9000
MSA-C vs. PD	0.8084	0.7798	0.7900	0.7841
MSA-C vs. PSP	0.7850	0.6870	0.7597	0.6539
PD vs. PSP	0.8564	0.8097	0.8145	0.7541

**Table A22:** MLP classifier training and testing balanced accuracy, sensitivity, and specificity using HC and T1w-only segmentation masks.

Differentiating Diseases	Train						Test					
	HC			T1w-Only			HC			T1w-Only		
	bAcc	Sen	Spe	bAcc	Sen	Spe	bAcc	Sen	Spe	bAcc	Sen	Spe
MSA-P vs. MSA-C	0.6173	0.7607	0.4738	0.5687	0.6483	0.4891	0.7645	0.8367	0.6922	0.7135	0.8134	0.6136
MSA-P vs. PD	0.8054	0.8361	0.7748	0.6748	0.7015	0.6481	0.8951	0.9073	0.8830	0.8800	0.8958	0.8642
MSA-P vs. PSP	0.6904	0.8071	0.5738	0.6786	0.8146	0.5426	0.6416	0.8138	0.4693	0.6128	0.8051	0.4205
MSA-C vs. PD	0.7680	0.7275	0.8084	0.7364	0.6929	0.7798	0.7707	0.7514	0.7900	0.7386	0.6932	0.7841
MSA-C vs. PSP	0.5938	0.6532	0.5343	0.5134	0.5657	0.4612	0.5476	0.6254	0.4698	0.5056	0.4102	0.6010
PD vs. PSP	0.6555	0.7990	0.5121	0.6426	0.7833	0.5019	0.6943	0.7969	0.5918	0.6826	0.8099	0.5552

bAcc: balanced accuracy, Sen: sensitivity, Spe: specificity.

**Table A23:** MLP classifier training and testing accuracy using HC and T1w-only segmentation masks.

Differentiating Diseases	Train		Test	
	HC	T1w-Only	HC	T1w-Only
MSA-P vs. MSA-C	0.7021	0.6444	0.7812	0.7575
MSA-P vs. PD	0.8153	0.7072	0.8875	0.8571
MSA-P vs. PSP	0.7886	0.7740	0.7890	0.7748
MSA-C vs. PD	0.7956	0.7685	0.7829	0.7725
MSA-C vs. PSP	0.6643	0.5643	0.6493	0.5172
PD vs. PSP	0.7705	0.7562	0.7551	0.7502

### Appendix C.7

The results of the classifier trained based on AdaBoost (ADA) are listed in Table A24, Table A25 and Table A26.

**Table A24:** ADA classifier training and testing AUC using HC and T1w-only segmentation masks.

Differentiating Diseases	Train		Test	
	HC	T1w-Only	HC	T1w-Only
MSA-P vs. MSA-C	0.8470	0.7486	0.8487	0.8287
MSA-P vs. PD	0.9079	0.8164	0.9021	0.8328
MSA-P vs. PSP	0.8697	0.8534	0.8426	0.8417
MSA-C vs. PD	0.7000	0.6536	0.6999	0.6743
MSA-C vs. PSP	0.9508	0.8884	0.9281	0.8939
PD vs. PSP	0.8789	0.7960	0.8806	0.8538

**Table A25:** ADA classifier training and testing balanced accuracy, sensitivity, and specificity using HC and T1w-only segmentation masks.

Differentiating Diseases	Train						Test					
	HC			T1w-Only			HC			T1w-Only		
	bAcc	Sen	Spe	bAcc	Sen	Spe	bAcc	Sen	Spe	bAcc	Sen	Spe
MSA-P vs. MSA-C	0.7619	0.8150	0.7088	0.6497	0.7368	0.5626	0.7486	0.8155	0.6816	0.7431	0.8158	0.6705
MSA-P vs. PD	0.8467	0.8163	0.8771	0.7562	0.6793	0.8330	0.8390	0.7969	0.8811	0.7770	0.7254	0.8286
MSA-P vs. PSP	0.7742	0.8378	0.7105	0.7450	0.8252	0.6648	0.7442	0.8369	0.6514	0.7275	0.8311	0.6239
MSA-C vs. PD	0.6421	0.4887	0.7956	0.6340	0.4683	0.7997	0.6632	0.5359	0.7906	0.6255	0.4586	0.7924
MSA-C vs. PSP	0.8725	0.8733	0.8716	0.8176	0.8336	0.8015	0.8656	0.8792	0.8521	0.8189	0.8321	0.8057
PD vs. PSP	0.7710	0.8879	0.6542	0.6861	0.8432	0.5290	0.7481	0.8743	0.6218	0.7143	0.8648	0.5638

bAcc: balanced accuracy, Sen: sensitivity, Spe: specificity.

**Table A26:** ADA classifier training and testing accuracy using HC and T1w-only segmentation masks.

Differentiating Diseases	Train		Test	
	HC	T1w-Only	HC	T1w-Only
MSA-P vs. MSA-C	0.77	0.6896	0.7529	0.7563
MSA-P vs. PD	0.8535	0.7760	0.85	0.7976
MSA-P vs. PSP	0.7960	0.7728	0.7690	0.7587
MSA-C vs. PD	0.7262	0.7254	0.7279	0.7145
MSA-C vs. PSP	0.8696	0.8062	0.8588	0.8094
PD vs. PSP	0.8333	0.7733	0.8176	0.8036

### Appendix C.8

Results of classifier using Gaussian naïve Bayes (GNB) are listed in Table A27, Table A28 and Table A29.

**Table A27:** GNB classifier training and testing AUC using HC and T1w-only segmentation masks.

Differentiating Diseases	Train		Test	
	HC	T1w-Only	HC	T1w-Only
MSA-P vs. MSA-C	0.8915	0.8397	0.8849	0.8683
MSA-P vs. PD	0.9279	0.8787	0.9245	0.8817
MSA-P vs. PSP	0.9054	0.8765	0.8961	0.8777
MSA-C vs. PD	0.7088	0.6610	0.7120	0.6652
MSA-C vs. PSP	0.9658	0.8840	0.9493	0.8721
PD vs. PSP	0.9198	0.8600	0.9154	0.8657

**Table A28:** GNB classifier training and testing balanced accuracy, sensitivity, and specificity using HC and T1w-only segmentation masks.

Differentiating Diseases	Train						Test					
	HC			T1w-Only			HC			T1w-Only		
	bAcc	Sen	Spe	bAcc	Sen	Spe	bAcc	Sen	Spe	bAcc	Sen	Spe
MSA-P vs. MSA-C	0.8417	0.9317	0.7517	0.7596	0.8622	0.6571	0.8225	0.9231	0.7218	0.8138	0.9066	0.7210
MSA-P vs. PD	0.8938	0.8860	0.9016	0.8434	0.7991	0.8876	0.8922	0.8713	0.9132	0.8073	0.7518	0.8629
MSA-P vs. PSP	0.7722	0.8789	0.6656	0.7670	0.9023	0.6317	0.7867	0.8889	0.6845	0.7626	0.8838	0.6413
MSA-C vs. PD	0.6767	0.5359	0.8175	0.6600	0.5099	0.8100	0.7179	0.6320	0.8039	0.6850	0.5569	0.8132
MSA-C vs. PSP	0.8806	0.9005	0.8607	0.7567	0.7839	0.7295	0.8383	0.8792	0.7974	0.7493	0.7840	0.7146
PD vs. PSP	0.7566	0.9218	0.5913	0.7179	0.8922	0.5435	0.7515	0.9353	0.5676	0.7083	0.9123	0.5042

bAcc: balanced accuracy, Sen: sensitivity, Spe: specificity.

**Table A29:** GNB classifier training and testing accuracy using HC and T1w-only segmentation masks.

Differentiating Diseases	Train		Test	
	HC	T1w-Only	HC	T1w-Only
MSA-P vs. MSA-C	0.8465	0.7705	0.8308	0.8272
MSA-P vs. PD	0.8948	0.8557	0.8964	0.8125
MSA-P vs. PSP	0.8030	0.7988	0.8135	0.7890
MSA-C vs. PD	0.7579	0.7322	0.7691	0.7562
MSA-C vs. PSP	0.8774	0.7556	0.8362	0.7581
PD vs. PSP	0.8162	0.7857	0.8133	0.7807

### Appendix C.9

Table A30, Table A31 and Table A32 list the results of the quadratic discriminant analysis (QDA) classifier.

**Table A30:** QDA classifier training and testing AUC using HC and T1w-only segmentation masks.

Differentiating Diseases	Train		Test	
	HC	T1w-Only	HC	T1w-Only
MSA-P vs. MSA-C	0.8953	0.8550	0.9029	0.8749
MSA-P vs. PD	0.9317	0.8898	0.9200	0.9000
MSA-P vs. PSP	0.8915	0.8929	0.9150	0.9091
MSA-C vs. PD	0.6925	0.6929	0.7457	0.6580
MSA-C vs. PSP	0.9368	0.8589	0.9056	0.8382
PD vs. PSP	0.8870	0.7944	0.8951	0.8638

**Table A31:** QDA classifier training and testing balanced accuracy, sensitivity, and specificity using HC and T1w-only segmentation masks.

Differentiating Diseases	Train						Test					
	HC			T1w-Only			HC			T1w-Only		
	bAcc	Sen	Spe	bAcc	Sen	Spe	bAcc	Sen	Spe	bAcc	Sen	Spe
MSA-P vs. MSA-C	0.7903	0.8005	0.7801	0.6847	0.7916	0.5778	0.8179	0.8595	0.7762	0.7937	0.8567	0.7307
MSA-P vs. PD	0.8818	0.8510	0.9126	0.8445	0.8005	0.8885	0.8804	0.8501	0.9107	0.8273	0.7688	0.8858
MSA-P vs. PSP	0.7996	0.7916	0.8076	0.7877	0.7975	0.7779	0.8381	0.8561	0.8201	0.8199	0.8655	0.7742
MSA-C vs. PD	0.7051	0.5971	0.8130	0.6555	0.5179	0.7932	0.7204	0.6295	0.8112	0.6669	0.5567	0.7772
MSA-C vs. PSP	0.8885	0.8281	0.9489	0.7630	0.7646	0.7614	0.7948	0.7871	0.8025	0.7655	0.7607	0.7704
PD vs. PSP	0.7868	0.8655	0.7081	0.7213	0.8434	0.5991	0.7841	0.8926	0.6755	0.7414	0.8892	0.5937

bAcc: balanced accuracy, Sen: sensitivity, Spe: specificity.

**Table A32:** QDA classifier training and testing accuracy using HC and T1w-only segmentation masks.

Differentiating Diseases	Train		Test	
	HC	T1w-Only	HC	T1w-Only
MSA-P vs. MSA-C	0.7921	0.7478	0.8266	0.8111
MSA-P vs. PD	0.8880	0.8564	0.8857	0.8392
MSA-P vs. PSP	0.7944	0.7816	0.8445	0.8374
MSA-C vs. PD	0.7706	0.7362	0.7741	0.7341
MSA-C vs. PSP	0.8479	0.7287	0.7812	0.7478
PD vs. PSP	0.8314	0.7933	0.8407	0.8180
